# Atypical imaging findings in a renal transplant patient with reversible posterior leukoencephalopathy syndrome: a case report

**DOI:** 10.1186/1757-1626-2-145

**Published:** 2009-09-30

**Authors:** Cristina Soler Riera, Leila Haddad, Darío Scocco, Gabriela Fischer, Christian Lopez Saubidet, Paulino A Álvarez

**Affiliations:** 1 Department of Internal Medicine, Centro de Educación Médica e Investigaciones Clínicas (CEMIC), Av Las Heras 2900 (C1425ASS), Buenos Aires, Argentina; 2 Department of Internal Medicine, Neurology Section, Centro de Educación Médica e Investigaciones Clínicas (CEMIC), Av Las Heras 2900 (C1425ASS), Buenos Aires, Argentina; 3 Department of Internal Medicine, Nephrology Section, Centro de Educación Médica e Investigaciones Clínicas (CEMIC), Av Las Heras 2900 (C1425ASS), Buenos Aires, Argentina

## Abstract

**Background:**

Atypical clinical and imaging findings in Reversible Posterior Leukoencephalopathy Syndrome are recognized with increasing frequency.

**Case report:**

We report a case of an adult in his 5^th ^decade immunosupressed with methilprednisolone, tacrolimus and micophenolate who two months after renal transplantation, multiple infections and an episode of humoral rejection became hypertensive with severe headaches, visual field abnormalities, seizures, left hemiparesis and hemineglect.

Computed Tomography scan of the brain showed a hypo dense lesion in the left occipital lobe. Ischemic stroke was diagnosed and aspirin and permissive hypertension were indicated. Twelve hours later he developed left sided motor seizures and cortical blindness. Magnetic Resonance Image showed hyper intensity in T2 and FLAIR in both occipital lobes and a small area of cortical restricted diffusion in Diffuson Weighted Images in the left occipital lobe. With a diagnosis of Reversible Posterior Leukoencephalopathy Syndrome his blood pressure was controlled with intravenous labetalol, and two days later the neurologic findings returned to baseline and most Computed tomography findings resolved.

**Conclusion:**

This case underscores that in the appropriate setting Reversible Posterior Leukoencephalopathy Syndrome should be suspected and the clinician should not be misled by atypical clinical or imaging findings. In contrast to other pathologies that resemble Reversible Posterior Leukoencephalopathy Syndrome, with the right and timely treatment, signs, symptoms and images can be completely reversible.

## Case Presentation

A 44-year-old caucasian man underwent his second kidney transplantation. His induction regimen included thymoglobulin, methilprednisolone and rituximab. Because of increased risk of humoral rejection he underwent 5 plasmapheresis sessions before transplant. He was 1.6 m tall, and weighted 70 kg.

The maintenance immunosuppressive regimen included methilprednisolone, tacrolimus and micophenolate. Delayed graft function occurred and a renal biopsy done in the first week after transplant showed signs of probable humoral rejection. Gammaglobulin and plasmapheresis were started with mild improvement of renal function.

His medical history includes chronic renal failure secondary to focal and segmental glomerulonephritis, first renal transplantation 23 years before the present admission and on dialysis for the last 10 years, hepatitis C and left eye blindness of unknown etiology. He had no relevant family history and did not smoke or drink alcohol.

During first two months his post-transplant course was complicated by surgical wound infection, urinary fistula, nosocomial pneumonia due to Haemophilus influenzae, and abdominal wall abscess due to Acinetobacter baumanii.

**Figure 1:**
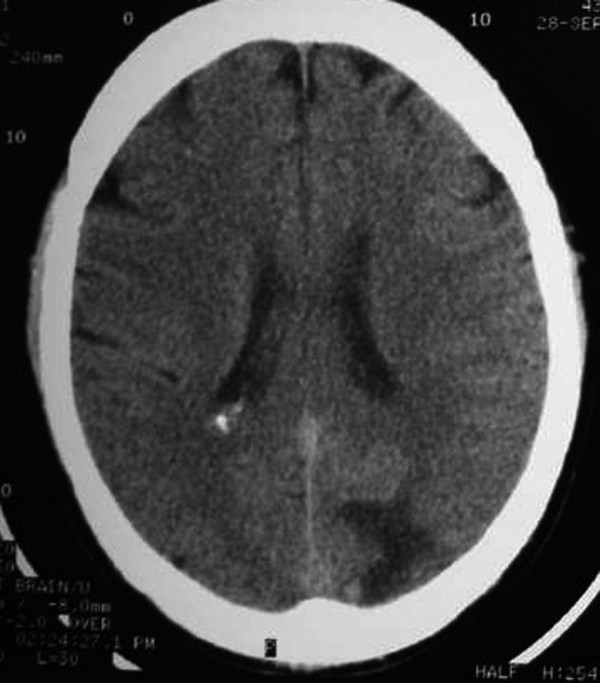
**Brain CT without contrast: left occipital cortico-subcortical hypodense area**.

One month after transplant the patient developed severe left sided headaches and blurred vision. He was hypertensive (150/90 mmHg with a usual blood pressure between 90-60 and 70-50) and a right temporal visual field deficit was detected. Computed Tomography (CT) showed a left occipital cortico-subcortical hypodense area. (Figure 1)

Serum creatinine was 3,79 mg/dl, tacrolimus level was 6.8 (normal 5-20), platelets were 137.000/ml. Other laboratory test results are shown in table 1.

**Table 1:** Laboratory test results

Variable	Reference range	28-9-2008	29-9-2008	30-9-2008	16-10-2008
Hematocrit	42-50%	34			34

Hemoglobin	14-17 g/dl	11			11.4

Platelets	150000-300000/ml	131000			131000

Leukocytes	4000 - 10000/ml	4800			6000

Na	136-145 mEq/L	136			138

K	3.5-5.1 mEq/L	5.5			4.7

Creatinine	0.7-1.2 mg/dl	3.79			3.25

BUN	3.73- 23.3 mg/dl	76.44			96.94

Bilirrubin	0.2-1.1 mg/dl	0.7			

Total Protein	6.4-8.3 gr/dl	4.4			

Quick	70-100%	89%			

APTT	30-45 sec	33			

Calcium	4.5- 5.5 mEq/L	3.8			4

TGP	10-35 U/L	14			

TGO	10-40 U/L	14			

Ph	7.37-7.43	7.28			7.37

pCO2	35-45 mmHg	37			25

HCO3-	24-26 milimoles/l	17.7			14

Mg	1.6-2.5 mg/dl			1.5	

Tacrolimus	5-20 nanograms/ml		6.8		

A Neurology consultant suggested ischemic stroke as the cause, aspirin was started and permissive hypertension was allowed.

**Figure 2:**
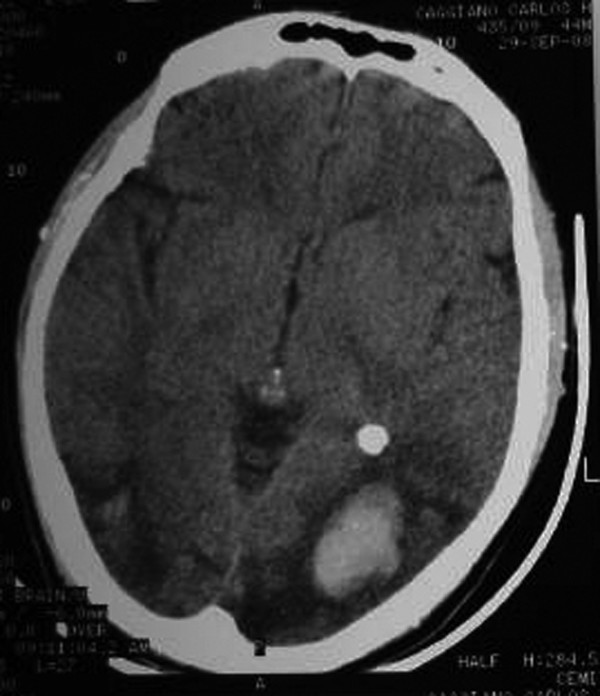
**Brain CT without contrast bilateral occipital cortico-subcortical hypodensities and hemorrhagic foci**.

**Figure 3:**
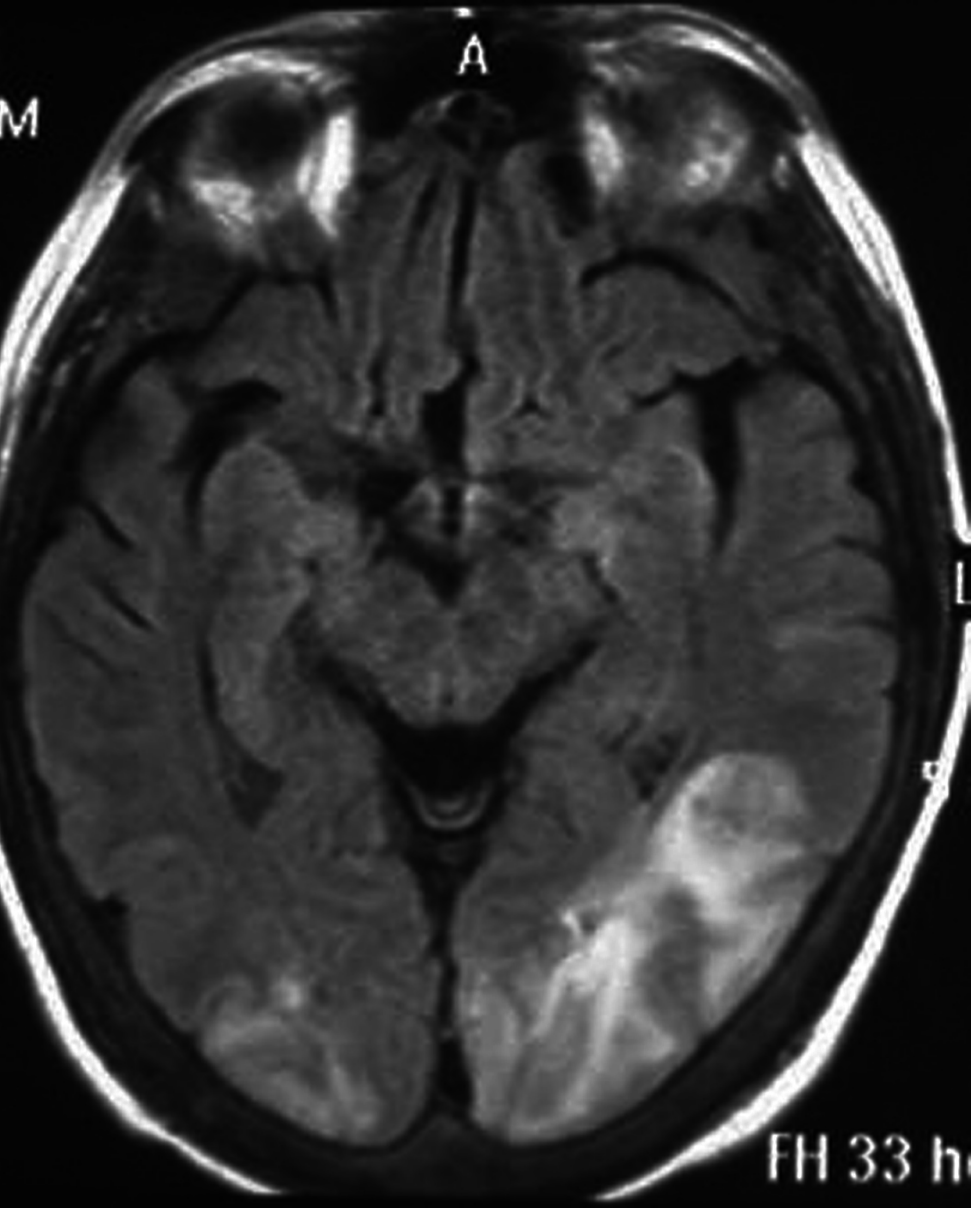
**Brain MRI FLAIR Bilateral parieto-occipital hyperintese lesions**.

**Figure 4:**
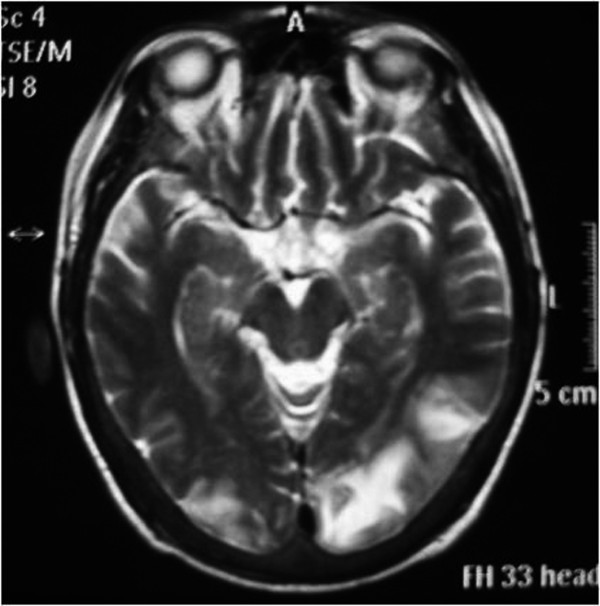
**Brain MRI T2 Bilateral parieto-occipital hyperintese lesion**.

**Figure 5:**
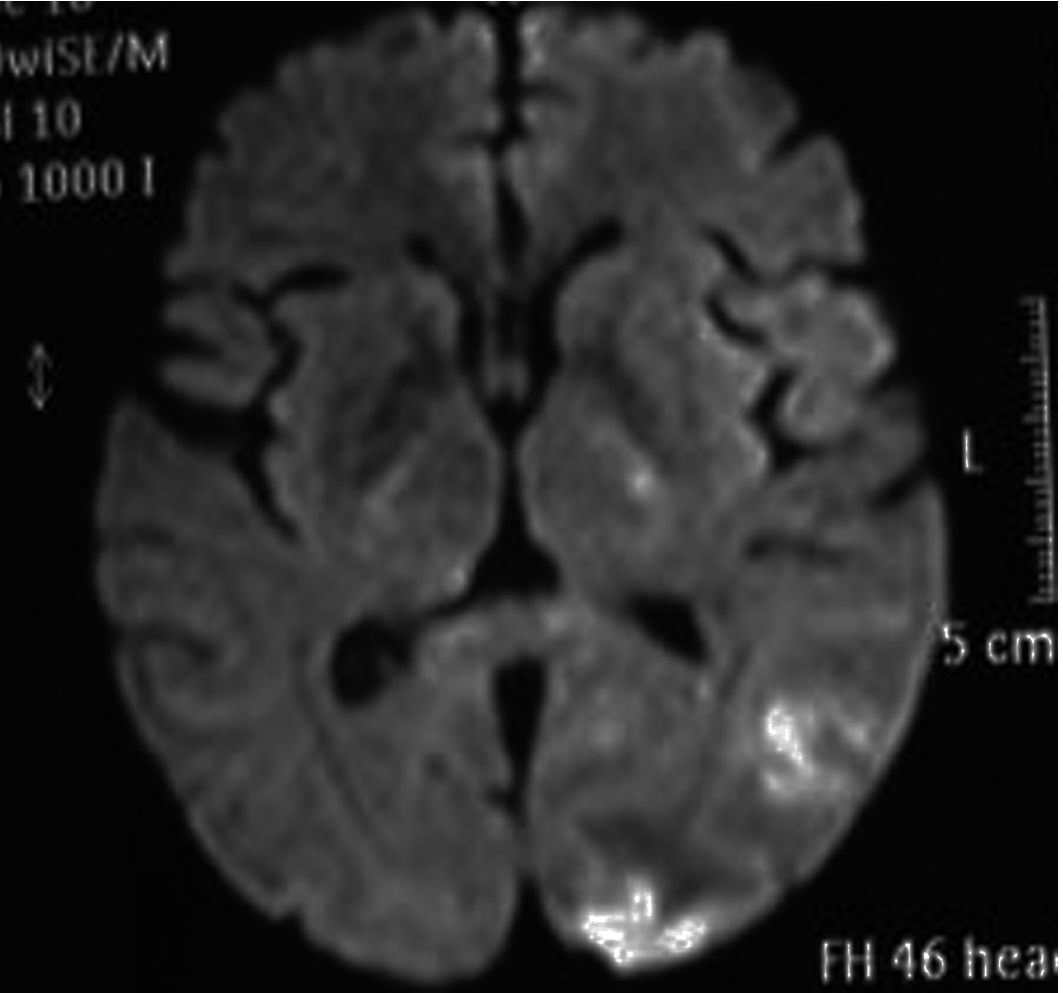
**Brain MRI DWI: left occipital cortical restriction in DWI**.

**Figure 6:**
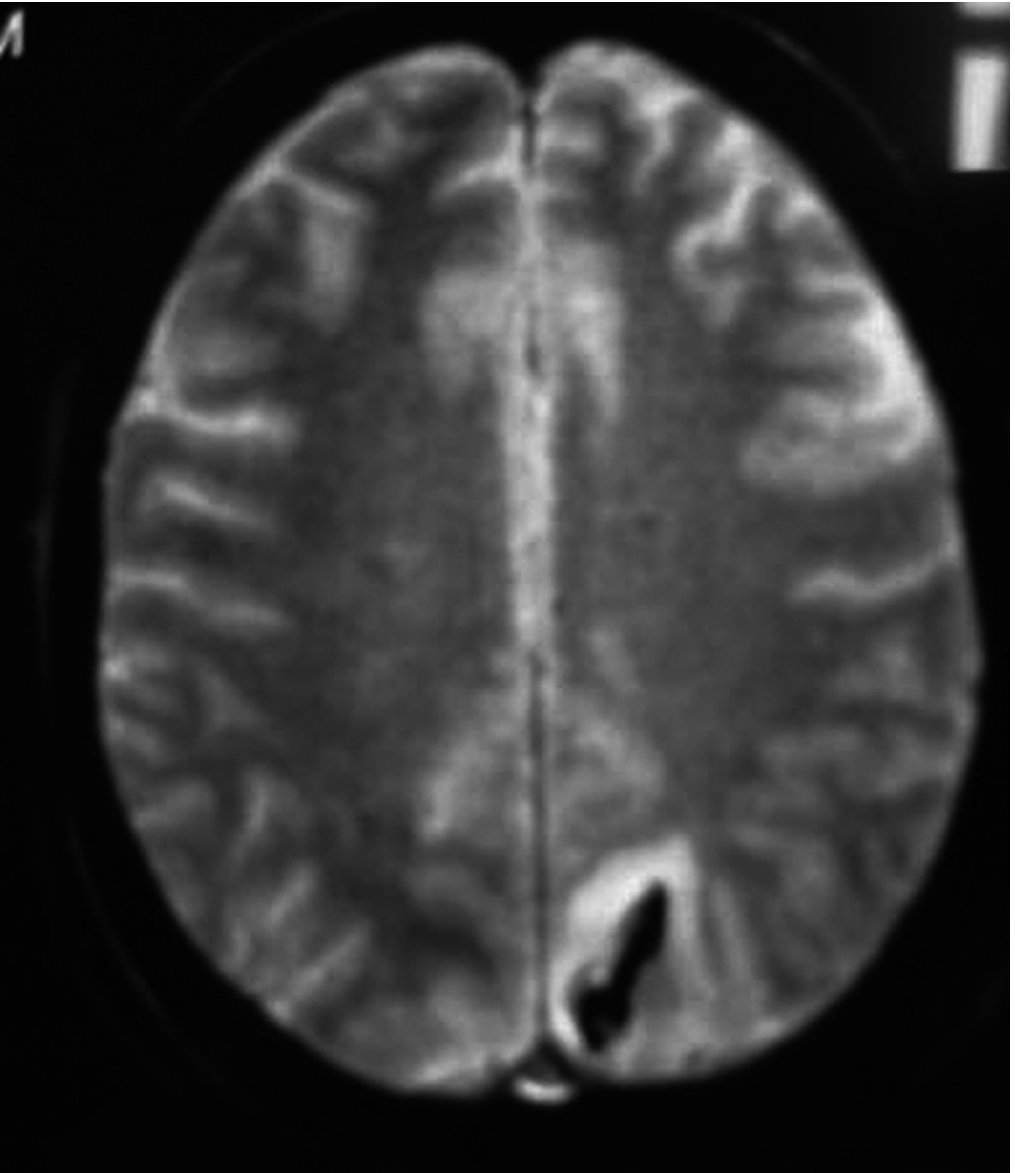
**Brain MRI GRE: left subcortical parieto-occipital hemorragic foci**.

**Figure 7:**
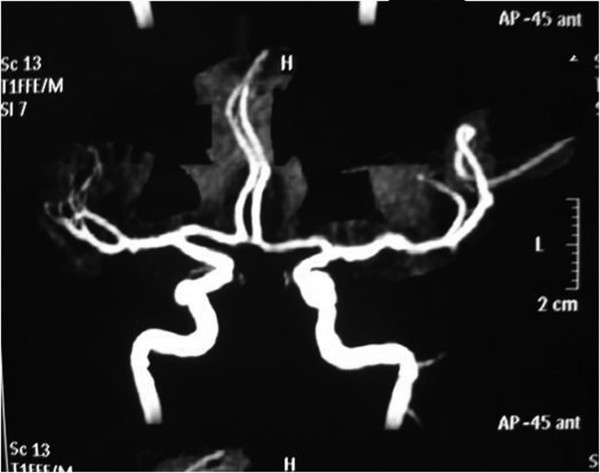
**Brain MRI Angiography**. Normal.

**Figure 8:**
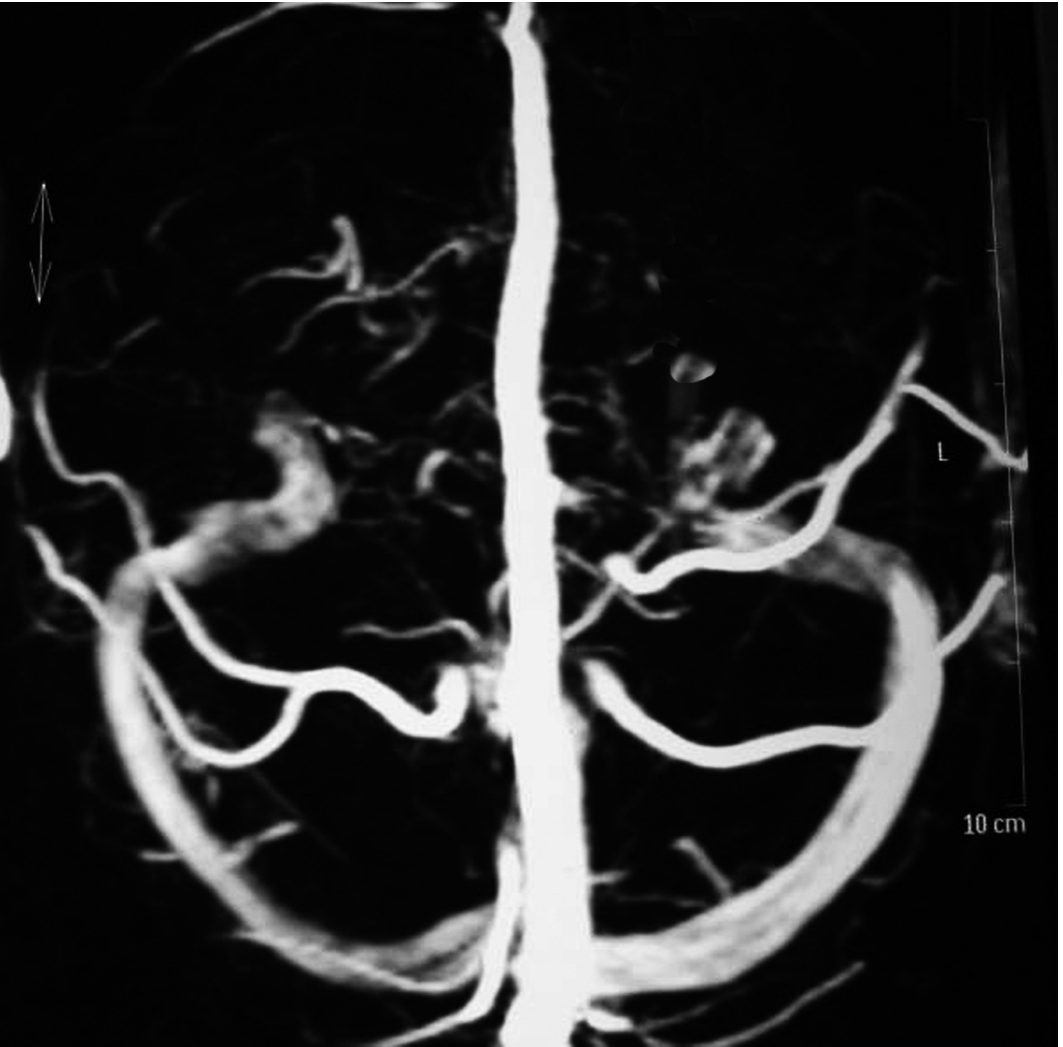
**Brain MRI Venous Angiography**. Normal.

Twelve hours later he had an episode of left-sided motor focal seizures followed by left hemiparesis and was admitted to the intensive care unit. He was alert and oriented. Blood pressure was 165/71, temperature 36.3°C, heart rate 88, pulse oximetry 100% on room air, respiratory rate 18. The neurological exam showed left arm and left leg weakness and bilateral amaurosis. A new CT scan showed bilateral occipital hypodensities and hemorrhagic foci. (Figure 2) Diphenilhydantoin was started. Brain Magnetic Resonance Image (MRI) showed bilateral occipital hyperintese lesions in Fluid Attenuated Inversion Recovery (FLAIR) and T2, a small area of cortical left occipital lobe restricted diffusion on Diffusion Weighted Image (DWI) and GRE sequence compatible with hemorrhage. (Figure 3, 4, 5, 6) MRI venous and arterial angiography showed no abnormalities. (Figure 7,8)

With a diagnosis of Reversible Posterior Leukoencephalopathy Syndrome (RPLS) blood pressure was controlled with intravenous labetalol (2 doses of 10 mg). Neurologic findings including cortical blindness returned to normal within 48 hs.

**Figure 9:**
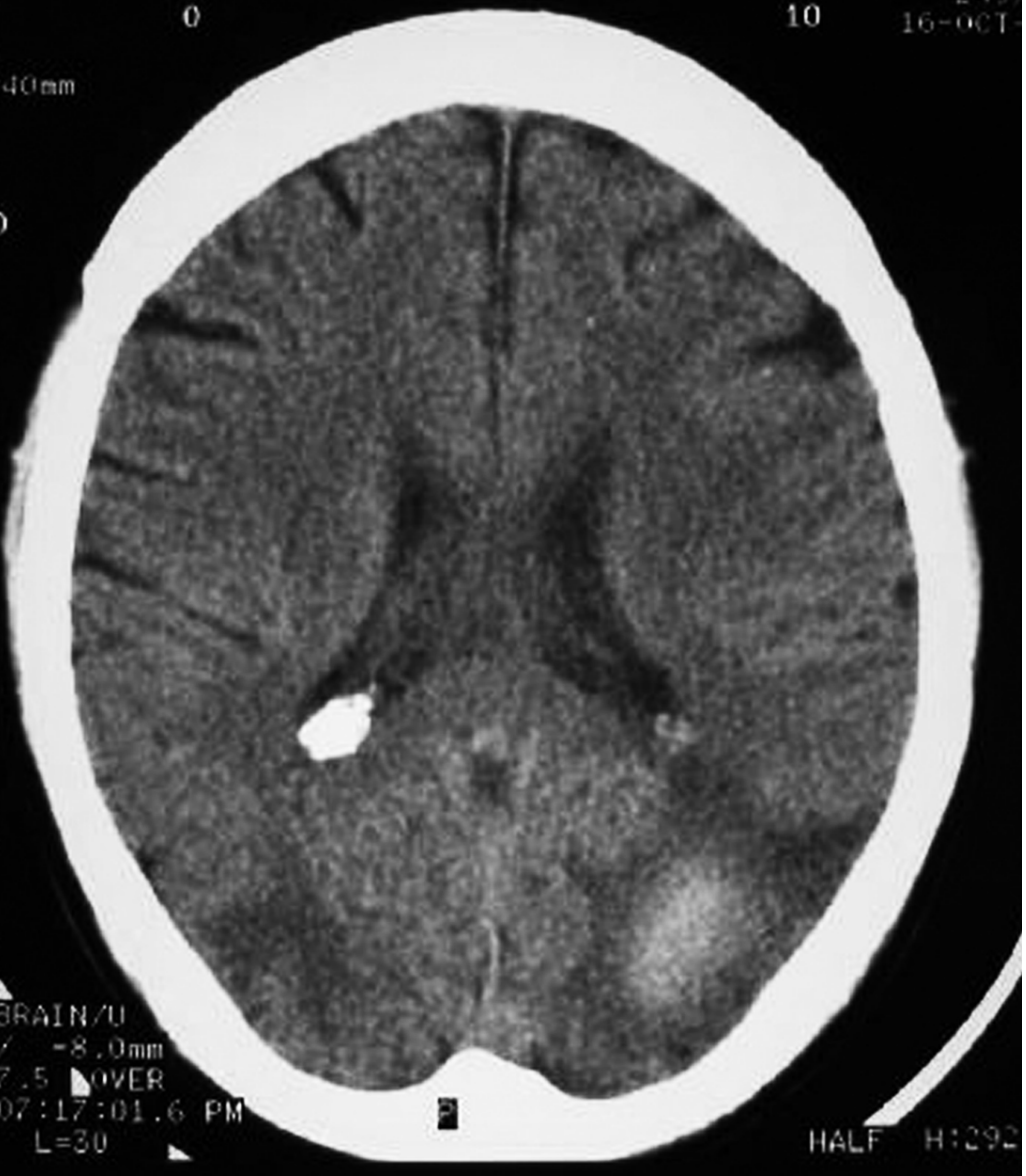
**Brain CT without contrast**. Decreasing hypodensity in left occipital and parietal lobes with improvement of hemorrhagic foci and almost complete resolution of right occipital lesions.

A follow-up CT 17 days later showed decreasing hypodensity in left occipital and parietal lobes with improvement of the hemorrhagic foci and almost complete resolution of the right occipital lesions. (Figure 9)

## Discussion

RPLS is a clinical radiologic syndrome of heterogeneous etiologies. In spite of its name the syndrome is not always reversible, and it is often not confined to either the white matter or the posterior regions of the brain [1]. Most common causes are hypertensive encephalopathy, ecclampsia, treatment with immunosuppressive agents or cytotoxic drugs and renal failure with hypertension. In solid organ transplantation the incidence of RPLS is low, between 0.49 and 6% [2].

Since the initial report in 1996 by Hinchey and coworkers the list of potential offending conditions have been increasing [3]. Although cyclosporine is the most common cytotoxic therapy associated with RPLS, it has been described with other agents including tacrolimus, sirolimus, cisplatin, interferon, and bevacizumab.

Among the many described causes of RPLS, hypertension, tacrolimus, immunoglobulin therapy and renal failure were present in our patient.

The pathogenesis of RPLS remains unclear, but it appears to be related to disordered cerebral autoregulation and endothelial dysfunction. Cytotoxic therapies have direct toxicity on vascular endothelium [1,3]. RPLS associated with these therapies may occur in normotensive individuals (although blood pressure is usually elevated over baseline), with nontoxic levels of these drugs, and it can happen even after several months of exposure to therapeutic levels [1,3,4].

The combination of acute hypertension and endothelial damage results in hydrostatic edema. Hyperperfusion and edema is mainly seen in the posterior circulation, perhaps because there is a greater concentration of adrenergic nerves (thought to be responsible for cerebral autoregulation) around pial and intracerebral vessels in the anterior circulation than posteriorly.

Common clinical manifestations include headaches, altered consciousness, visual disturbances, and seizures.

The classic neuroimaging feature is edema involving the white matter in the posterior portions of the cerebral hemispheres, usually bilaterally in the parieto-occipital regions. The lesions are seen as white-matter hypodensities on CT scans and hypointense in T1, hyperintense in T2 and in FLAIR on MRI studies [5,6]. Atypical distributions and imaging manifestations such as significant anterior involvement, cortical lesions, recurrent RPLS episodes, foci of permanent injury, hemorrhage into lesions, unilaterality, and areas of restricted diffusion are described with increasing frequency in recent and larger series of patients [7,8]. Diffusion restriction in RPLS has been shown to occur in a minority of patients (17% in a series) and usually is punctuate, small, or patchy and is surrounded by much larger areas of vasogenic edema [8,6].

Differential diagnosis include stroke, venous thrombosis, toxic or metabolic encephalopathy, demyelinating disorders, vasculitis, and encephalitis among others. Particularly in cases with a sudden onset of neurologic symptoms, as was the case of our patient, the presentation can mimic bilateral posterior cerebral artery infarctions ("top of the basilar syndrome"). The fact that RPLS usually spares the calcarine and paramedian occipital lobe helps to differentiate these two entities [5]. This distinction is important because in RPLS hypertension should be treated in order to prevent permanent brain injury and blood pressure management in ischemic stroke remains controversial [9]. Furthermore RPLS patients should not receive antiplatelet treatment.

## Conclusion

This reports calls attention to the fact that in the appropriate setting RPLS should be suspected and the clinician should not be misled by atypical clinical or imaging findings. In contrast to other pathologies that resemble RPLS, prompt recognition and treatment may prevent permanent damage.

## Consent

Written informed consent was obtained from the patient for publication of this case report and accompanying images. A copy of the written consent is available for review by the Editor-in-Chief of this journal.

## Competing interests

The authors declare that they have no competing interests.

## Authors' contributions

PA, CS, CLS and LH analyzed and interpreted the patient clinical data and wrote the manuscript.DS and GF interpreted data regarding neurological and nephrological disease respectively. All authors read and approved the final manuscript.
